# Combined Transcriptomics and Metabolomics Identify Regulatory Mechanisms of Porcine Vertebral Chondrocyte Development In Vitro

**DOI:** 10.3390/ijms25021189

**Published:** 2024-01-18

**Authors:** Mingming Xue, Ning Huang, Yabiao Luo, Xiaoyang Yang, Yubei Wang, Meiying Fang

**Affiliations:** 1 Department of Animal Genetics and Breeding, National Engineering Laboratory for Animal Breeding, MOA Key Laboratory of Animal Genetics and Breeding, Beijing Key Laboratory for Animal Genetic Improvement, State Key Laboratory of Animal Biotech Breeding, Frontiers Science Center for Molecular Design Breeding, College of Animal Science and Technology, China Agricultural University, Beijing 100193, China; mingmxue@163.com (M.X.); yabiaoluo2021@163.com (Y.L.); yangxy95v1@163.com (X.Y.); 2 Sanya Research Institute, China Agricultural University, Sanya 572025, China; 15390751795@163.com (N.H.); pq007007@hotmail.com (Y.W.)

**Keywords:** pig, chondrocytes, transcriptome, energy metabolome, transcriptional regulation

## Abstract

Porcine body length is closely related to meat production, growth, and reproductive performance, thus playing a key role in the profitability of the pork industry. Cartilage development is critical to longitudinal elongation of individual vertebrae. This study isolated primary porcine vertebral chondrocytes (PVCs) to clarify the complex mechanisms of elongation. We used transcriptome and target energy metabolome technologies to confirm crucial genes and metabolites in primary PVCs at different differentiation stages (0, 4, 8, and 12 days). Pairwise comparisons of the four stages identified 4566 differentially expressed genes (DEGs). Time-series gene cluster and functional analyses of these DEGs revealed four clusters related to metabolic processes, cartilage development, vascular development, and cell cycle regulation. We constructed a transcriptional regulatory network determining chondrocyte maturation. The network indicated that significantly enriched transcription factor (TF) families, including zf-C2H2, homeobox, TF_bZIP, and RHD, are important in cell cycle and differentiation processes. Further, dynamic network biomarker (DNB) analysis revealed that day 4 was the tipping point for chondrocyte development, consistent with morphological and metabolic changes. We found 24 DNB DEGs, including the TFs *NFATC2* and *SP7.* Targeted energy metabolome analysis showed that most metabolites were elevated throughout chondrocyte development; notably, 16 differentially regulated metabolites (DRMs) were increased at three time points after cell differentiation. In conclusion, integrated metabolome and transcriptome analyses highlighted the importance of amino acid biosynthesis in chondrocyte development, with coordinated regulation of DEGs and DRMs promoting PVC differentiation via glucose oxidation. These findings reveal the regulatory mechanisms underlying PVC development and provide an important theoretical reference for improving pork production.

## 1. Introduction

In pig breeding, body size is a major selection criterion, being closely associated with growth rate, feed efficiency, reproductive performance, and lifespan. Body length is a common metric for assessing body size in pigs and other domestic animals, strongly correlated with meat production and a key indicator of enhanced livestock performance. Porcine body length can be increased through augmenting the vertebrae number or elongating individual vertebrae, with the former being more common in breeding programs. However, variations in vertebral count represent only a small fraction of the phenotypic variation in body length [1]. Therefore, understanding the development of individual vertebrae is crucial for improving pork output.

Spinal vertebral bones develop through endochondral ossification, a process involving differentiation of mesenchymal cells to form chondrocytes, agglutination to form cartilage models of future bone, chondrocyte maturation and mineralization, and the formation of ossification centers. Stimulating chondrocytes into a hypertrophic state is critical for driving longitudinal vertebral elongation [2,3]. Various molecular elements regulate endochondral ossification, including transcription factors (TFs) such as *SOX9* and *RUNX2* [4,5,6], along with signaling pathways such as Indian hedgehog (Ihh), BMP, and Notch [7,8,9,10]. Their dysregulation can impair chondrogenesis and cause skeletal dysplasia.

Previously, we identified the porcine TF *NKX3.2*, a key regulator of chondrogenic differentiation, and confirmed *NKX3.2* differential expression in vertebral column development via transcriptomic analysis of thoracic intervertebral cartilage from Yorkshire and Wuzhishan pigs at 1 and 4 months [11]. The rapid development of transcriptomics has opened new possibilities for further exploration of porcine cartilage development, facilitating the discovery of additional crucial factors and unraveling complex mechanisms.

In addition to the involvement of TFs, energy metabolism plays a major role in endochondral ossification [12]. Chondrocytes rely on glycolysis to maintain energy homeostasis in an environment lacking vasculature and oxygen. As such, they exhibit the Warburg effect, or the ability to engage in high levels of glycolysis even when oxygen is present. Inadequate glycolysis causes an energy deficit that limits cell proliferation, activates incorrect protein folding, and decreases collagen synthesis [13]. Glucose metabolism is vital to endochondral ossification and cartilage development because of its relevance to cellular activity and cartilage matrix homeostasis [14,15,16]. Interference with glucose transport or metabolism genes alters chondrocyte maturation and skeletal development [17,18,19,20].

Although the function of energy metabolism during cartilage development is evident, the fate of relevant metabolites is poorly characterized, despite their importance during chondrogenesis [21,22,23,24]. Metabolomics investigates the composition of metabolites in the cells and tissues of biological systems [25]. Because metabolomics detects the end-products of biological processes, the results reflect the functional state and mechanisms of a system [26]. Previous studies have employed untargeted metabolomics to screen endogenous molecules involved in chondrogenic fate determination via biochemical–ecological niche control [27]. Therefore, metabolomics is a feasible suite of techniques for identifying key cartilage development pathways and metabolites.

This study aimed to investigate the mRNA expression profile of primary porcine vertebral chondrocytes (PVCs) at different stages of differentiation (0, 4, 8, and 12 days). Using these data, our goal was to understand variations in TF-regulated genes and identify those key to regulating chondrogenesis. We used dynamic network biomarkers (DNBs) to determine the tipping point of porcine chondrocyte differentiation and identify core networks that regulate developmental transitions during this process. Next, we applied targeted energy metabolomics to analyze the metabolic characteristics of developing PVCs. Through integrating differentially expressed genes (DEGs), differentially regulated metabolites (DRMs), and significant pathways, we elucidated the complex mechanisms underlying porcine vertebral cartilage development. The results should provide much-needed insights into longitudinal extension of the spine to enhance pork production.

## 2. Results

### 2.1. Identification of PVCs

**Figure 1:**
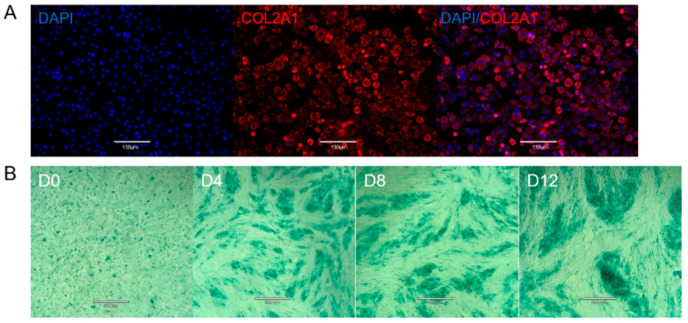
Identification of primary porcine vertebral chondrocytes (PVCs). (**A**) Immunofluoresence of primary PVCs. (**B**) Alcian blue staining plots of PVCs on days 0, 4, 8, and 12 of differentiation.

Within 24 h of isolation, the PVCs adhered to the culture dish, forming irregular, polygonal, or round shapes with an uneven distribution (Supplementary Figure S1). Initially, their growth rate was slow, but subsequent passages revealed consistent cell morphology (Supplementary Figure S1). Immunofluorescence staining revealed that almost all passage 1 (P1) PVCs were positive for COL2A1 (Figure 1A). Upon inducing differentiation, the PVCs transformed into larger, round cells, forming localized clusters (Supplementary Figure S1). Extending the induction duration led to deeper Alcian blue staining with greater cell clustering (Figure 1B).

### 2.2. Temporal Transcriptomic Characterization of PVC Development

**Figure 2:**
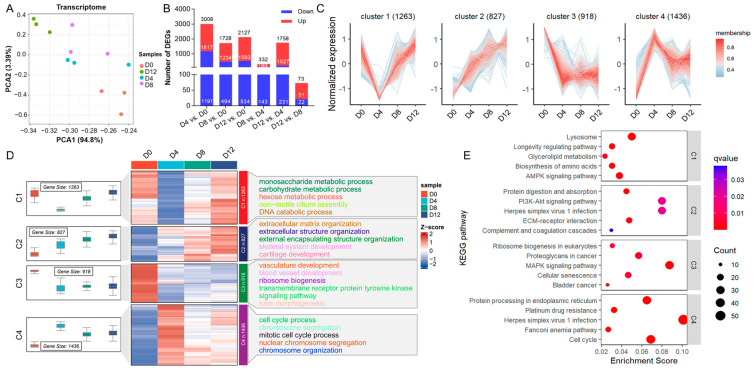
Characteristics of mRNA expression profiles of PVCs at various differentiation time points. (**A**) PCA of 12 samples, based on the normalized expression abondance of all the expressed mRNAs. (**B**) Summary of the DEG count in any two comparison groups. (**C**) MFUZZ time-series clustering of all DEGs (clusters = 4). (**D**) Heatmap of four clusters at four time points and top five significantly enriched GO terms for each cluster. (**E**) Top five significantly enriched KEGG pathways for each cluster.

We obtained 79.54 G of raw data from 12 libraries, separated into four groups named D0, D4, D8, and D12 (n = 3). The cleaned samples achieved a read score Q30 of 97% and GC content of no less than 45% (Supplementary Table S1). The PCA results indicated high similarity within groups (Figure 2A). Pairwise comparisons revealed 4566 DEGs across the four groups (Figure 2B, Supplementary Table S2). The expression patterns of the seven genes were quantified against the RNA-seq data, indicating that the RNA-seq results were accurate and reliable (Supplementary Figure S2). Time-series gene clustering analysis categorized the differentially expressed genes into four clusters (C1–C4) (Figure 2C, Supplementary Table S3). During pig chondrocyte development, the expression levels of 1263 DEGs associated with metabolic processes exhibited an initial decrease, followed by a subsequent increase in C1. The expression levels of 827 DEGs related to cartilage development showed a gradual increase with C2 expression. Additionally, the expression levels of 918 DEGs associated with vasculature development gradually increased at the C3 stage. Furthermore, the expression levels of 1436 DEGs related to cell cycle regulation showed an initial increase followed by a subsequent decrease in the C4 group (Figure 2D). The top five significantly enriched pathways in each cluster were identified (Figure 2E).

### 2.3. Characterizing Transcriptional Reprogramming during PVC Development

**Figure 3:**
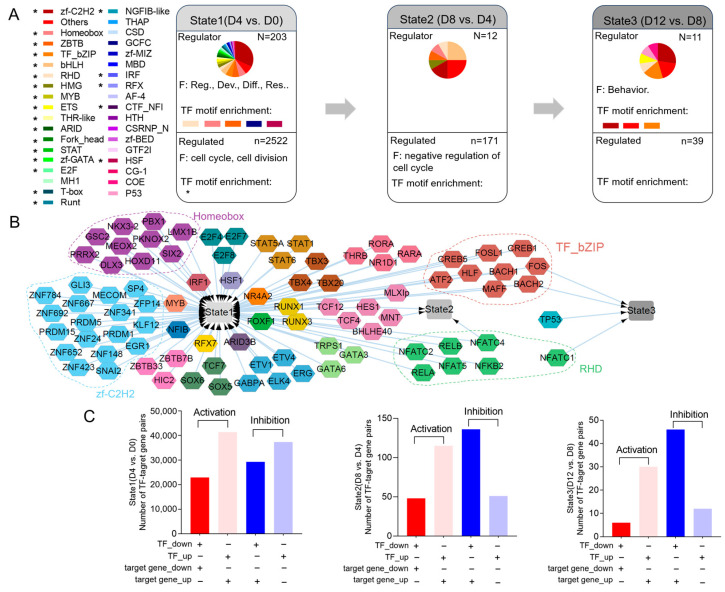
Schedule of chondrocyte developmental transcription reprogramming in PVCs. (**A**) Analysis of major transcriptional states in DEG regulatory network during PVC differentiation (states = 3). “N” represents the number of differentially expressed TFs, “n” represents the number of DEGs regulated by TFs. “F” represents gene function in GO enrichment analysis. Reg., regulation; Dev., development; Diff., differentiation; Res., response. (**B**) Directional regulation of transcriptional states in PVC development. (**C**) Number of transcriptional regulatory relationship pairs. Regulation (activation or inhibition) of TFs is determined by the relative change in expression of both the TFs and target genes between the two compared groups.

The schedule of transcriptional states during PVC development was determined via mining transcriptome data. We compared each stage of PVC development with previous stages and divided the DEGs into three gene sets to construct transcriptional regulatory states (states 1–3). To investigate the regulatory roles of each DEG set and their biological significance, we performed transcriptional regulation classification and functional enrichment of genes (Figure 3A, Supplementary Table S4). For instance, the first wave of transcriptional changes in state 1 was characterized by 203 TFs associated with regulation, development, and cell differentiation, as well as 2522 genes related to the cell cycle and cell division. State 1 was the most enriched DNA motif and included bHLH, homeobox, ZBTB, GCFC, and HSF.

To predict the directional interactions between the regulatory DEGs and regulated DEGs in each transcriptional state, we used TF DNA-binding motif data to establish the relationships between differentially expressed TFs and regulated DEGs. Furthermore, we established the gene regulatory network (GRN) in the three transcriptional states (Figure 3B). In this network, the largest number of TFs were activated to regulate state 1. The TF families, including zf-C2H2, homeobox, TF_bZIP, and RHD, were the most active because they had the largest TF count in transcriptional state 1. *HES1*, *FOS*, *BACH2*, and *NFATC4* were involved in more than one transcriptional state. Notably, the total number of target DEGs increased with transcriptional activation and inhibition was the largest in each comparison group, consistent with the largest number of increased DEGs (Figure 1B and Figure 3C, Supplementary Table S5).

### 2.4. Dynamic Gene Expression Landscape of PVC Development

**Figure 4:**
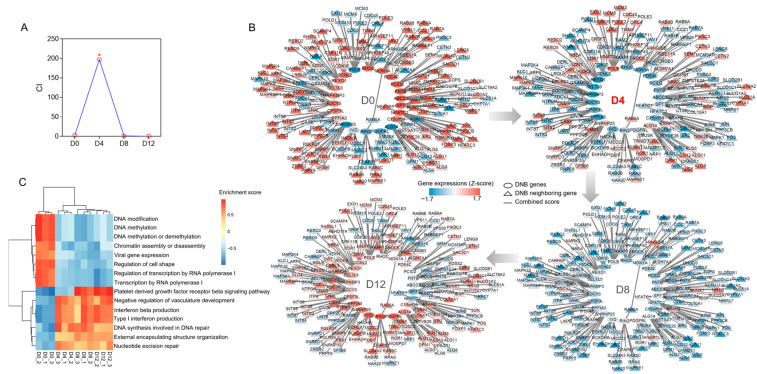
Dynamic network analysis of PVC development. (**A**) Criticality index (CI) scores at each time point of PVC development. The score was higher for D4 than for other time points, indicating that D4 was the differentiation tipping point. The asterisk (*) indicates the peak value. (**B**) Dynamic expression of DNBs and adjacent DEGs at different developmental stages. (**C**) Gene set variation analysis of all genes in the DNB member-centered network. Enrichment scores of the top 15 significant GO terms were analyzed for each sample based on the gene-set expression levels for each term.

We used DNB theory to derive the critical tipping point during chondrogenesis and identify the transition-specific genes involved. Applying the transcriptome atlas of PVCs at different developmental stages, we found that the DNB peak appeared on D4 and identified 24 genes crucial to the CI score as DNB members (Figure 4A, Supplementary Table S6). Based on molecular interactions from STRING, 22 DEGs encoding DNB-member proteins and their adjacent 183 DEGs were scanned and integrated to construct a DNB member-centered network (Figure 4B). The expression levels of all genes in this network changed before and after the critical point in PVC development.

Next, GSVA revealed that these genes were significantly enriched in GO terms related to transcriptional regulation, DNA synthesis and modification, vascular development, structural organization, and the immune system (Figure 4C). The enrichment scores of terms related to DNA modification and transcriptional regulation decreased with the differentiation time, whereas the enrichment scores of terms associated with the structural organization and negative regulation of vasculature development increased.

### 2.5. NFATC2 and SP7 Promote PVC Differentiation

**Figure 5:**
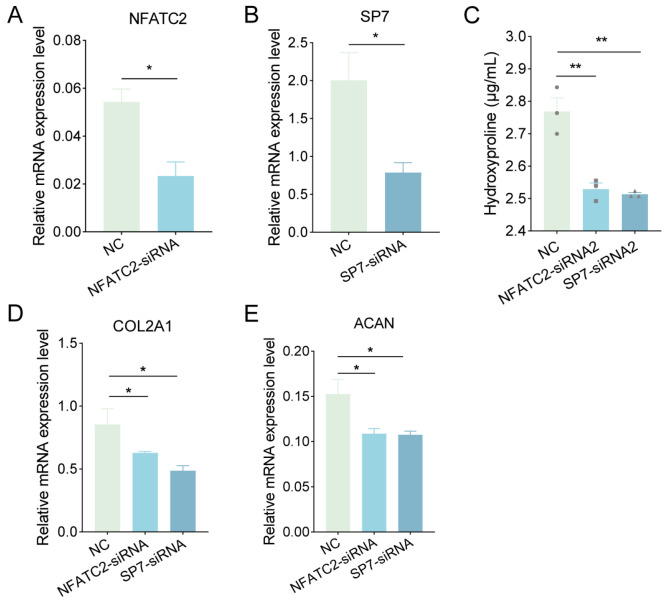
Effect of *NFATC2* or *SP7* knockdown on PVC development. PVCs were treated with *NFATC2* or *SP7* siRNAs or NC (negative control). After 48 h, cells were collected to detect their mRNA expression levels of (**A**) *NFATC2*, (**B**) *SP7*, (**D**) *COL2A1*, and (**E**) *ACAN*. (**C**) After 4 days, the cell supernatant was collected for hydroxyproline content analysis. * *p* < 0.05, ** *p* < 0.01.

DNB analysis identified *NFATC2* and *SP7* transcription factors as crucial regulators in PVC development. To explore their role, we utilized siRNAs to inhibit the mRNA expression levels of *NFATC2* or *SP7* (Figure 5A,B). The suppression of *NFATC2* or *SP7* resulted in a notable decrease in proteoglycan production, evident from the reduced hydroxyproline levels (Figure 5C). Moreover, silencing *NFATC2* or *SP7* significantly impeded the gene expression of chondrocyte markers, namely collagen type II alpha 1 chain (*COL2A1*) and aggrecan (*ACAN*) (Figure 5D,E), indicating a delay of PVC differentiation. Collectively, these data suggest that *NFATC2* and *SP7* play a promotive role in PCV differentiation.

### 2.6. Targeted Energy Metabolomic Analysis of PVC Development

**Figure 6:**
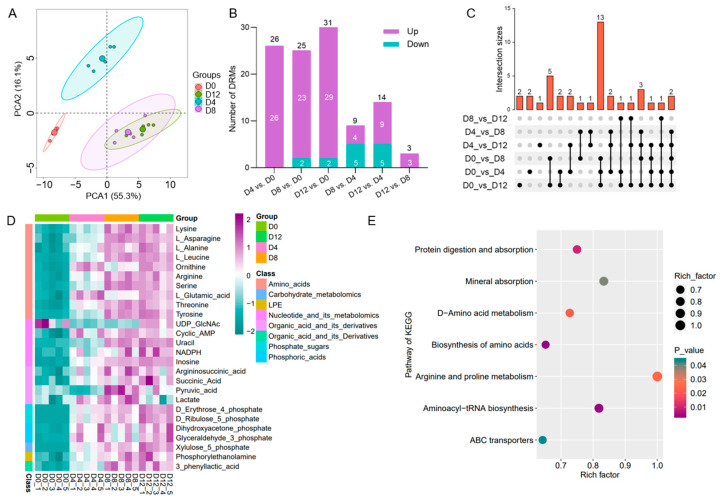
Characteristics of PVC energy metabolomics at various time points during differentiation. (**A**) PCA of all metabolites in the energy metabolome. (**B**) Summary of the DRM count across any two comparison groups. (**C**) UpSet plot of six comparison sets, displaying the number of common and unique DEGs. (**D**) Heatmap of 26 DRMs at four time points of PVC differentiation, based on the metabolite content of 20 samples. (**E**) KEGG enrichment analysis of 26 DRMs.

The PCA results of the UPLC-MS/MS data on four chondrocyte differentiation days (D0, D4, D8, D12) showed that the samples split into three clusters, with the D8 and D12 samples failing to separate (Figure 6A). Pairwise comparisons revealed that most DRMs were increased (Figure 6B). Compared with D0, 16 DRMs were consistently higher on D4, D8, and D12 (Figure 6C, Supplementary Table S7). We detected 47 metabolites, with 26 being significantly regulated in all four groups (Figure 6D, Supplementary Table S8). Interestingly, all DRMs except UDP_GlcNAc and lactate were increased during PVC differentiation. This increase in metabolites accounted for 95.7% of the total upregulation, suggesting that energy metabolism-related metabolites accumulated during chondrocyte differentiation (Figure 6D). KEGG analysis of the DRMs revealed seven significant pathways related to amino acid metabolism, transport, and utilization, including amino acid biosynthesis, aminoacyl-tRNA biosynthesis, protein digestion and absorption, d-amino acid metabolism, arginine and proline metabolism, mineral absorption, and ABC transporters (Figure 6E).

### 2.7. Integrated Analysis of DEG–DRM Co-Regulatory Mechanism in PVC Development

Our transcriptome and metabolomic analyses of the PVCs’ development demonstrated that DEGs and DRMs were significantly enriched in the same KEGG pathways, including amino acid biosynthesis, protein digestion, and absorption. Significantly enriched amino acids in the amino acid biosynthesis pathway included lysine, L-asparagine, L-alanine, L-leucine, ornithine, arginine, serine, L-glutamic acid (Glu), threonine, tyrosine, argininosuccinic acid, pyruvic acid, D-erythrose-4-phosphate, D-ribulose-5-phosphate, and dihydroxyacetone phosphate. In order to evaluate the impact of the amino acid biosynthesis pathway on chondrocyte development, the PVCs were subjected to treatment with 50 μM of Glu. Comparative analysis with the vehicle group revealed a significant increase in hydroxyproline levels in the Glu-treated group (Supplementary Figure S3A). Moreover, Glu at 50 μM induced a significant upregulation in the relative mRNA expression of key markers such as *COL2A1* and *SOX9*, indicating a promotive effect on PVC development (Supplementary Figure S3B,C).

**Figure 7:**
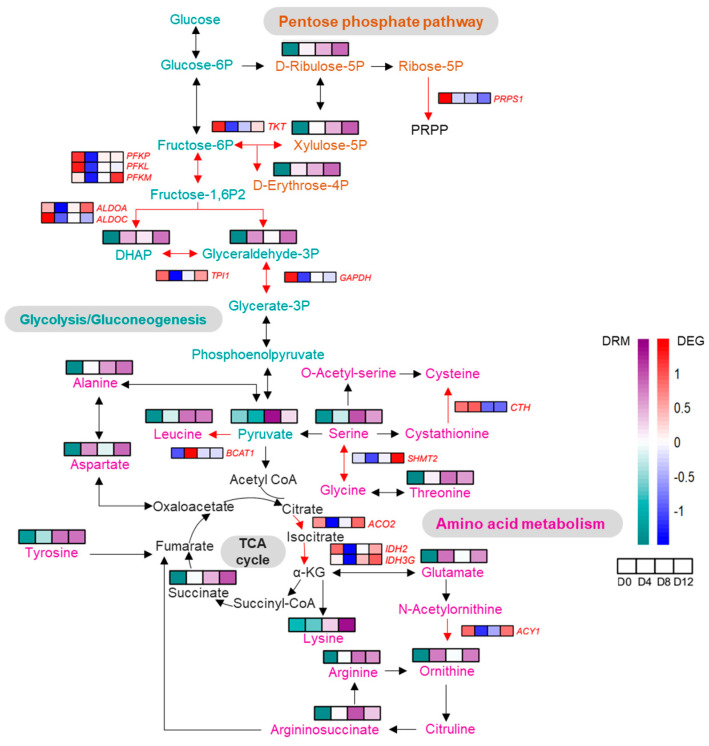
Comprehensive analysis of DEGs and DRMs involved in the KEGG amino acid biosynthesis pathway during PVC development.

To better understand the interactions between DEGs and variation in metabolic pathways during PVC development, we also mapped DEG–DRM regulatory processes, including glucose metabolism, amino acid metabolism, and the TCA cycle (Figure 7). Notably, these metabolic processes together comprised the “biosynthesis of amino acids” pathway, indicating that DEGs and DRMs act synergistically during chondrocyte differentiation. In the pentose phosphate and amino acid metabolism pathways, metabolites are converted into one other. This process appears to be only partially dependent on variations in the expression of associated enzyme-encoding genes. Overall, metabolite levels were not synchronized with changes in the abundance of enzyme-encoding genes.

## 3. Discussion

The vertebrate spine is derived from the somites of the embryonic paraxial mesoderm [28]. Body axis elongation and segmentation are major morphogenetic events that occur simultaneously during vertebrate embryonic development [29]. An individual vertebral body is formed through endochondral ossification, developing from cartilage templates. This process involves key steps such as mesenchymal condensation, chondrocyte differentiation and maturation, and osteoblast development [30]. Previous studies in pigs have shown that longer vertebral bodies have larger volumes of hypertrophic chondrocytes, indicating that chondrocyte development affects vertebral elongation [11]. Following from our existing body of knowledge, we isolated primary chondrocytes from porcine vertebral scleromeres, then performed transcriptomic and metabolomic analyses to clarify the molecular regulation underlying cartilage development. Our results indicated that almost all primary porcine chondrocytes positively expressed the cartilage marker gene *COL2A1*. Additionally, the chondrocytes exhibited a swirling arrangement and deeper blue color from Alcian blue staining [31] as they differentiated, confirming the success of our porcine chondrocyte model.

Transcriptome analysis is an effective approach for studying spatiotemporal gene expression and the biological functions associated with co-expressed genes. Previous studies using RNA sequencing have examined gene expression patterns and key regulators of chondrogenesis in chicken embryonic limb bud-derived progenitors, providing a transcriptomic signature that controls chondrogenesis [32]. In this study, we constructed a time-series gene expression landscape for PVC development to analyze the relevant physiological activities and gene mechanisms. Our time-series gene clustering categorized DEGs into four clusters (C1–C4), with C1 regulating metabolic processes. Functional analysis indicated that the C1 DEGs were significantly enriched in the AMPK signaling pathway and amino acid biosynthesis (Figure 2E). AMPK signaling regulates different metabolic processes and serves as a key hub for energy sensing and metabolic regulation [33]. Next, the DEGs in C2 were associated with cartilage development, and their expression in extracellular structures peaked on day 12, coinciding with the deepest Alcian blue staining (Figure 1B and Figure 2D). The C3 DEGs are related to vascular development and were decreased during differentiation, suggesting that osteogenesis was inhibited during the induction of chondrocyte hypertrophy. Local vasculature in the skeletal system is essential to and actively participates in bone formation [34]. Finally, the C4 DEGs are involved in the cell cycle and exhibited their highest expression on day 4, followed by a decrease, reflecting cell cycle arrest before chondrocyte hypertrophy [30]. Taken together, our data suggest that chondrocyte development is governed by a combination of extracellular structural organization, cell cycle processes, vasculature development, and metabolic processes.

The activity of TFs influences all stages of cellular activity, including differentiation, fate, and signaling. Transcription factors have been proposed as “engines” of bone elongation, determining and modulating the genetic program of chondrocyte differentiation and maturation [35]. Based on the schedule of chondrocyte developmental transcriptional reprogramming, state 1 (D4 vs. D0) showed the highest TF enrichment, suggesting that the main regulatory stage of cell differentiation occurs during early chondrocyte development (Figure 3). Consistent with our findings on transcriptional reprogramming regulation, DNB analysis showed that day 4 of cell differentiation was a key transition point in PVC development (Figure 4A). Target DEGs regulated by TFs were enriched in the cell cycle, implying that TFs are crucial to chondrocyte development via their cell cycle regulation (Figure 3A). In state 1, TFs *NKX3-2*, *GLI3*, *FOS*, and *RELA* modulate chondrocyte differentiation and viability, according to our data and those of previous reports (Figure 3B) [36,37,38,39,40,41]. *GLI3* is also important to bone development as a key effector of Ihh activity, acting as an osteogenic regulator of vasculature-derived signaling and as a determinant of mesenchymal progenitor cell fate [42]. Our experiments further demonstrated that *HES1*, *FOS*, *BACH2*, and *NFATC4* were involved in more than one transcriptional regulatory state (Figure 3B). Previous research has revealed that RBPjκ-dependent Notch targets *HES1* to promote the onset of chondrocyte hypertrophy [43]. Finally, we uncovered several novel regulatory functions of TFs in chondrocyte differentiation, notably the actions of *NFATC2* and *SP7* in dynamic gene expression during PVC development (Figure 4B). The suppression of *NFATC2* and *SP7* led to a marked inhibition of proteoglycan production and a decrease in chondrogenic gene expression levels (Figure 5). Overall, the specific roles of other TFs in chondrocyte differentiation warrant further investigation.

The DNB method has been successfully applied to many questions involving animal and plant development, identifying early signals of critical transitions and their core regulators based on omics data and nonlinear dynamical theory [44,45,46]. Here, we established a dynamic DNB member-centered network that linked DNB genes with their neighboring genes for four stages of PVC development (Figure 4B). This network contained 24 DNB molecules that are key regulators of PVC development. The results of our GSVA confirmed these DNB findings and highlighted the major biological processes that were dependent on specific stages in PVC development (Figure 4C). Further, DNB analysis uncovered that day 4 marked a critical juncture. Concurrently, the PVCs exhibited noticeable proteoglycan on day 4 of the cell differentiation process (Figure 1B). Notably, the number of differentially expressed genes and metabolites was highest from day 0 to day 4, followed by day 4 to day 8, and day 8 to day 12 during the continuous differentiation of PVCs. These findings strongly suggest that a significant shift in the regulation of chondrogenesis occurs early in the differentiation process.

The extracellular matrix of chondrocytes serves as a scaffold for cellular structural organization, providing signals for proliferation and differentiation that are essential for normal endochondral ossification [30,47]. In this study, we identified significant alterations in amino acid and glucose metabolism, partly reflecting the responses of intracellular metabolites to porcine chondrogenic differentiation and maturation. A previous study showed that aspartate supports chondrocyte proliferation and matrix synthesis, while glutathione (from glutamine catabolism) prevents the accumulation of intracellular reactive oxygen species and is beneficial for maintaining chondrocytic redox homeostasis [21]. Here, we showed that during PVC differentiation, Alcian blue staining gradually deepens for chondrocytes (Figure 1B). Moreover, amino acid levels were significantly increased; in particular, L-asparagine and L-glutamic acid concentrations were high during chondrocyte differentiation, indicating that their increase contributed to protein and extracellular matrix synthesis (Figure 5D,E).

In an avascular environment, chondrocytes rely on the relatively inefficient glycolysis for metabolic energy. Hence, amino acid upregulation during chondrogenic differentiation may serve as a compensatory mechanism by providing additional biomolecules [12,13,16]. In our integrated metabolomics and transcriptomics analysis, the abundance of DEGs encoding glycolysis- and tricarboxylic acid cycle-related enzymes decreased significantly during the early stages of PVC differentiation (Figure 6). This decrease was consistent with the overall abundance of DEGs that regulated metabolic processes in C1, followed by a subsequent recovery (Figure 2D). Conversely, a large number of DRMs were elevated throughout chondrocyte development (Figure 5D).

Metabolites and genes are intricately linked, as metabolite levels and compositions can act as signaling molecules or co-regulators of gene expression, while the products of gene expression generate metabolites [26]. This complex relationship poses interpretative challenges for analyses based solely on gene expression. For instance, our omics data clearly revealed a non-linear relationship between DRM levels and the abundance of metabolic regulatory DEGs. Therefore, we strongly recommend that future studies employ single-factor experimental designs to further explore the role of specific metabolites. In our study, differential metabolites and genes jointly regulated amino acid synthesis, with disturbances observed in the glucose oxidation and pentose phosphate pathways (Figure 5E and Figure 6). Despite these disruptions, the chondrocytes maintained optimal energy homeostasis, as evidenced by the absence of significant changes in ATP levels (Supplementary Tables S7 and S8). These findings highlight the crucial role of the interplay between DRMs and DEGs in overall metabolic control and adaptability during PVC development.

## 4. Materials and Methods

### 4.1. Isolation and Culture of Primary Porcine Vertebral Chondrocytes (PVCs)

Using enzyme digestion, the PVCs were isolated from cartilage tissues close to the vertebral segmentation joint of a large neonatal white pig. Cartilage samples were dissected into millimeter-sized pieces and then incubated for 8 h in DMEM/F12 supplemented with 2% fetal bovine serum (FBS), 3% penicillin-streptomycin, 1% amphotericin B, and 0.2% collagenase II at 37 °C. The single-cell suspension was filtered through a 40 μm nylon cell strainer, and then centrifuged at 300× *g* for 10 min. Subsequently, cells were collected and cultured (37 °C, 5% CO_2_) in DMEM/F12 supplemented with 10% FBS and 1% penicillin-streptomycin. To induce chondrogenic differentiation, subconfluent cultures were incubated in DMEM/F12 containing 5% FBS, 1% amphotericin B, and 1% insulin-transferrin-selenium (Gibco, Grand Island, NY, USA) [48]. The cell culture medium was replaced every 2 days.

### 4.2. Immunofluorescence

Primary PVCs were fixed in 4% paraformaldehyde (Solarbio, Beijing, China) for 30 min at 25 °C. Cells were then permeabilized with 1% Triton X-100 solution for 15 min and blocked with an immunostaining blocking buffer for 15 min. Subsequently, the cells were incubated with primary antibody against COL2A1 (1:200, BOSTER, Wuhan, China) at 4 °C overnight, and with anti-rabbit fluorescent secondary antibody conjugated to CoraLite594 (1:500, Proteintech, Wuhan, China) at 37 °C for 60 min. Nuclei were stained with DAPI (Beyotime, Shanghai, China). Cells were photographed under a fluorescence microscope (Echo Laboratories, San Diego, CA, USA).

### 4.3. Alcian Blue Staining Assay

Cells were washed twice with PBS, fixed with 4% paraformaldehyde at room temperature for 30 min, and stained with Alcian blue cartilage stain solution (PH1.0, Solarbio, China) for 30 min. Samples were rinsed thrice with PBS and imaged.

### 4.4. Cell Culture and Transfection

For the Glu supplement assay, PVCs were seeded in 12-well plates and cultured until reaching approximately 100% confluence. Subsequently, the PVCs were exposed to a differentiation medium supplemented with 50 μM Glu (Sigma-Aldrich, St. Louis, MO, USA) or a control vehicle.

For gene knockdown experiments, PVCs were seeded in plates and cultured until reaching approximately 80% confluence. RNA fragments, including *NFATC2* siRNA, *SP7* siRNA, and a negative control (NC), were transfected into the PVCs using GP-transfect-mate (GenePharma, Shanghai, China), following the manufacturer’s instructions.

### 4.5. Hydroxyproline Assay

PVC supernatant was collected to detect the hydroxyproline content using a hydroxyproline assay kit (Nanjing jiancheng, Nanjing, China), according to the manufacturer’s protocols. Absorbance was determined at 550 nm.

### 4.6. Transcriptomic Analysis

Cells were separately harvested on days 0 (D0), 4 (D4), 8 (D8), and 12 (D12) of differentiation. Total RNA was extracted with TRIzol reagent, then quantified and quality-checked using a Nanodrop. Twelve cDNA libraries were synthesized from four groups (n = 3) using SuperScript™ II Reverse Transcriptase (Invitrogen, Waltham, MA, USA), and then subjected to pairwise sequencing on an Illumina HiSeq Novaseq™ 6000 platform. Sequenced data were quality-controlled, filtered, and mapped to the pig reference genome (Sus *scrofa11.1*, GenBank Assembly Accession: GCF _00003025.6) in HISAT2. Gene expression was calculated in feature counts and normalized according to the number of transcripts per million mapped reads (FPKM). Differential gene expression analysis was performed using the limma package in R, employing a linear model based on empirical Bayesian methods. The threshold for DEGs was set as *p* < 0.05 and |log_2_fold change| ≥ 1.

### 4.7. cDNA Synthesis and Quantitative Real-Time PCR

Seven DEGs (*CCDC80*, *COL14A1*, *ITGBL1*, *MATN2*, *NFATC2*, *SP7*, and *PAPSS2*) were randomly selected for quantitative real-time PCR (qRT-PCR) validation. Complementary DNA was synthesized using FastKing gDNA Dispelling RT SuperMix (TIANGEN, Beijing, China), following the manufacturer’s protocol. The reaction was performed using Taq Pro Universal SYBR qPCR Master Mix (Vazyme, Nanjing, China) and primers designed with NCBI Primer-BLAST. The thermocycling process (Bio-Rad CFX96 Real-Time PCR system) adhered to the following schedule: 95 °C for 30 s, 40 cycles of 95 °C for 10 s and 60 °C for 30 s, then a final melting/dissociation curve stage. Porcine *RN18S* was selected as the internal control [49]. Relative expression was calculated using the 2^−ΔΔCt^ method. For primer sequences, see Supplementary Table S9.

### 4.8. Time-Series Gene Clustering and Functional Analysis

The fuzzy c-means algorithm was applied for the clustering and visualization of time-series DEG data in the R package ClusterGVis. Functional analysis (Gene Ontology, GO; Kyoto Encyclopedia of Genes and Genomes, KEGG) of the DEGs was conducted in the R package clusterProfiler [50].

### 4.9. Motif Analysis

Differentially expressed TFs at the contiguous developmental stage and their families were identified in AnimalTFDB version 4.0 [51]. A cumulative hypergeometric distribution with the total TF families as the background was applied to analyze TF enrichment within a given gene list. Significant enrichment was determined using the Fisher’s exact test. High-quality TF-binding motifs within 1 kb upstream of DEGs were scanned, and significantly enriched motifs were identified using the FIMO tool [52]. Relationships between regulator TFs and their target genes were also determined. Finally, TF regulation in each developmental state was visualized in Cytoscape.

### 4.10. Analysis of DNBs

The nonlinear dynamical theory of cell development posits that analyses of DNBs can identify the critical tipping point of cell development [53]. Therefore, gene expression data across all four chondrocyte developmental stages were analyzed using the DNB package in R [54]. The protein-coding genes of DNB members were mapped onto the protein–protein interaction network from the STRING database.

In the DNB analysis, observed data must satisfy three statistical conditions. First, the gene expression levels among DNB members should fluctuate widely, with an increased standard deviation (SDin). Second, correlations between the DNB members should increase dramatically, as evidenced by high absolute Pearson correlation coefficients (PCCin). Third, associations between DNB members and other genes should decrease, characterized by low absolute Pearson correlation coefficients (PCCout). These conditions result in the criticality index (CI), as follows:

$$\mathrm{CI}=\frac{\mathrm{PCCin}}{\mathrm{PCCout}}\mathrm{SDin}$$

When the CI peaks during this period, the biological system reaches a critical tipping point.

### 4.11. Gene Set Variation Analysis (GSVA)

Biological processes at different PVC developmental stages were identified through GSVA, a nonparametric, unsupervised method for detecting small changes in pathway activity among a large number of gene sets [55]. The gene set of GO terms from the Molecular Signatures Database (MsigDB) was extracted using the R msigdbr package [56]. The GSVA package in R was used to calculate the enrichment score of GO terms in each sample [57]. The Benjamini–Hochberg correction method for multiple comparisons was applied to our GO analyses. Significant GO terms were those with adjusted *p* ≤ 0.05.

### 4.12. Target Energy Metabolomics

Cell samples were collected at D0, D4, D8, and D12 of differentiation and stored at −80 °C for targeted energy metabolomics analysis. After the samples were thawed on ice, 500 μL of 80% methanol was added and the mixture was vortexed. Subsequently, the samples were subjected to three freeze–thaw cycles before the supernatant was collected and filtered through a protein precipitation plate. Quality control (QC) samples were prepared via mixing equal aliquots of supernatant from all samples. Analysis was performed using ultra-performance liquid chromatography coupled with tandem mass spectrometry (UPLC–MS/MS).

Liquid chromatography was performed using ACQUITY UPLC BEH Amide columns (1.7 µm, 100 mm × 2.1 mm i.d.) under the following conditions: flow rate = 0.40 mL/min, column temperature = 40 °C, and injection volume = 2 μL. The mobile phase consisted of A (ultrapure water, 10 mM ammonium acetate, and 0.3% ammonia) and B (90% acetonitrile). The gradient elution procedure was as follows: 0–1.2 min A/B at 5:95 (*v*/*v*), 8 min A/B at 30:70 (*v*/*v*), 9.0–11 min A/B at 50:50 (*v*/*v*), and 11.1–15 min A/B at 5:95 (*v*/*v*). Parameters of the Q-Trap 6500+ for MS were as follows: electrospray ionization temperature, 550 °C; MS voltage, 5500 V in positive ion mode and −4500 V in negative ion mode; curtain gas pressure, 35 psi.

Chromatographic peak areas and retention times from the MS data were extracted in MultiQuant 3.0.3. Standard curves were established for 64 metabolites to enable qualitative and quantitative analyses. Utilizing the Orthogonal Partial Least Squares Discriminant Analysis (OPLS-DA) model, Variable Importance in Projection (VIP) was employed for the initial screening of metabolites showing differences between different groups. Screening of DRMs between any two groups used the thresholds of fold change ≤ 0.05 or ≥2 and VIP > 1. Multiple groups were compared with ANOVA, and DRMs were identified via the filtering criteria of *p* < 0.05 and VIP > 1.

### 4.13. Statistical Analysis

Statistical analysis was conducted using SPSS 25.0 software (SPSS Inc., Chicago, IL, USA). A one-way analysis of variance followed by Duncan’s multiple range test was employed for data evaluation. Statistical significance was established at a threshold of *p* < 0.05.

## 5. Conclusions

In this study, we analyzed the characteristics of gene regulatory networks and cellular metabolism during PVC development. Our analysis revealed that 4566 DEGs collectively regulated chondrocyte development through four processes: metabolism, cartilage development, vascular development, and the cell cycle. We also established a gene regulatory network related to PVC development and a DNB member-centered network, identifying key TFs *NFATC2* and *SP7* that regulate the initiation of PVC differentiation. Additionally, our integrated omics analysis emphasizes the significance of amino acid biosynthesis in chondrocyte development, highlighting the coordinated regulation of DEGs and DRMs in promoting PVC differentiation via glucose oxidation. Therefore, our findings provide a better understanding of the complex regulatory mechanisms underlying PVC development, offering valuable molecular data for improving porcine body length traits. This study also serves as a crucial reference for increasing pork production by improving the length of porcine vertebrae.

## Data Availability

The RNA-seq datasets have been deposited in the BIG Data Center of the Beijing Institute of Genomics, Chinese Academy of Sciences, and they are publicly accessible at https://ngdc.cncb.ac.cn/gsa (CRA012775). Accessed on 19 September 2025.

## Supplementary Materials

The following supporting information can be downloaded at: https://www.mdpi.com/article/10.3390/ijms25021189/s1.
