# Supplementary figures and images for: Combined Transcriptomics and Metabolomics Identify Regulatory Mechanisms of Porcine Vertebral Chondrocyte Development In Vitro
Source: Int J Mol Sci. 2024 Jan 18;25(2):1189. doi: 10.3390/ijms25021189 (PMC10816887; doi:10.3390/ijms25021189)

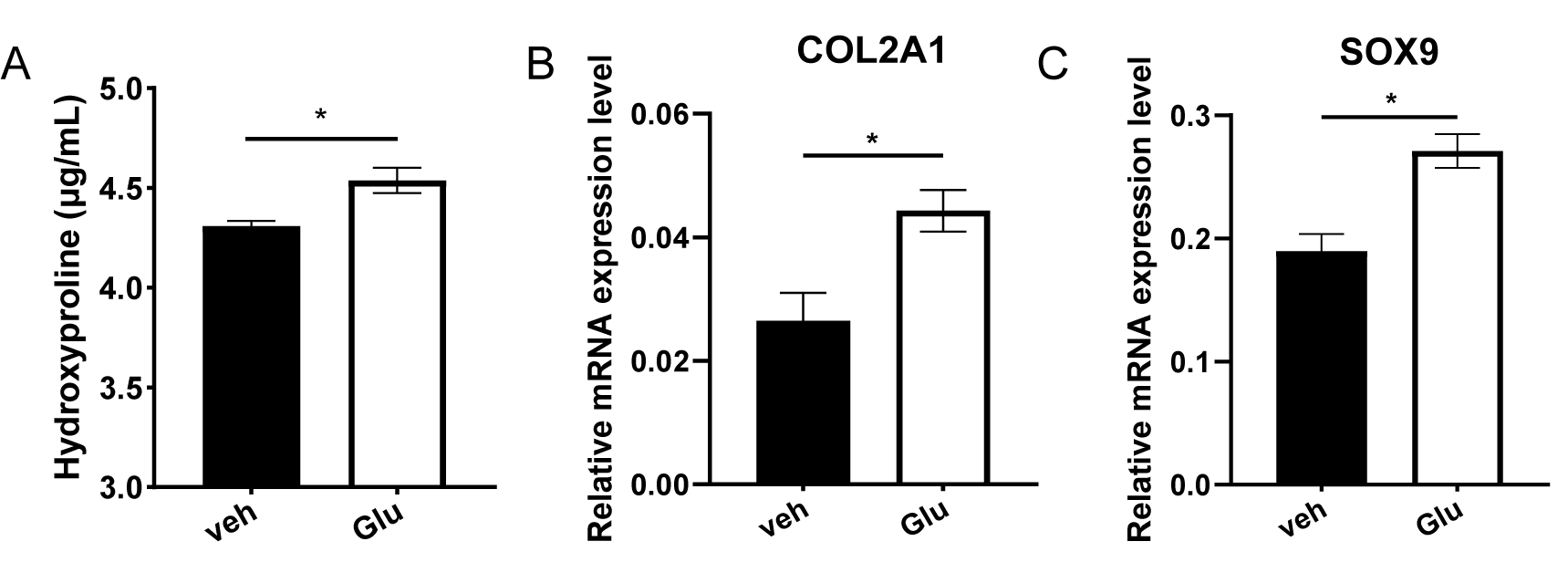

Supplement: Supplementary file 1 [file ijms-25-01189-s001.zip › Figure S3.tif]
